# Materials for Direct Air Capture and Integrated CO_2_ Conversion: Advancement, Challenges, and Prospects

**DOI:** 10.1021/acsmaterialsau.3c00061

**Published:** 2023-08-31

**Authors:** Marcileia Zanatta

**Affiliations:** † Institute of Advanced Materials (INAM), Universitat Jaume I, Avda Sos Baynat s/n, 12071 Castellón, Spain

**Keywords:** CO_2_ capture, CO_2_ reuse, sorption, catalysis, atmospheric air

## Abstract

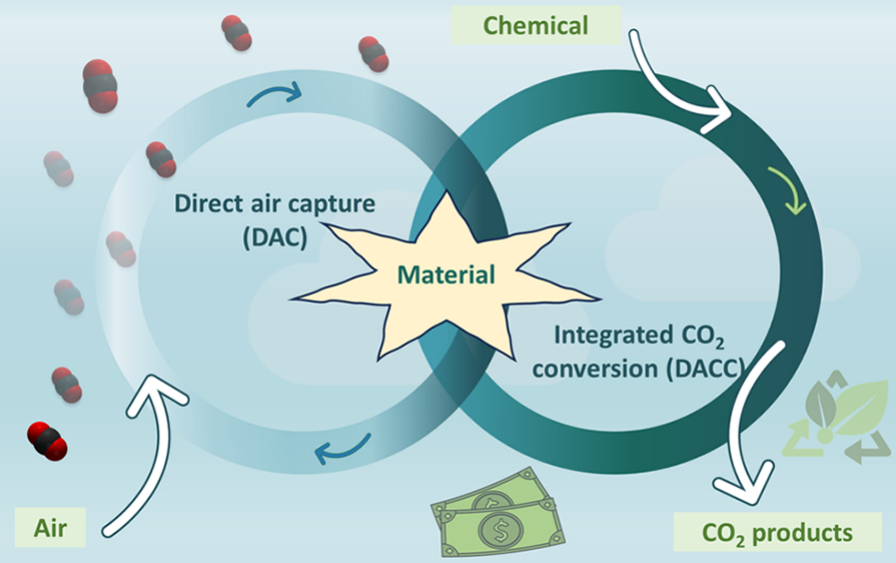

Direct air capture and integrated CO_2_ conversion (DACC) technologies have emerged as promising approaches to mitigate the increasing concentration of carbon dioxide (CO_2_) in the Earth’s atmosphere. This Perspective provides a comprehensive overview of recent advancements in materials for capturing and converting atmospheric CO_2_. It highlights the crucial role of materials in achieving efficient and selective CO_2_ capture as well as catalysts for CO_2_ conversion. The paper discusses the performance, limitations, and prospects of various materials in the context of sustainable CO_2_ mitigation strategies. Furthermore, it explores the multiple roles DACC can play in stabilizing atmospheric CO_2_.

## 1 Introduction

Direct air capture (DAC) is a technology that aims to capture carbon dioxide (CO_2_) directly from the atmosphere. The rising levels of CO_2_ contribute significantly to climate change and global warming. Addressing the impacts of these emissions requires effective strategies for both capturing and converting CO_2_. The approach of direct air capture and its subsequent conversion (DACC) into value-added products has emerged as a promising solution to this global issue.^1−3^

Materials play a crucial role in the development of efficient DACC systems.^4^ These materials serve as catalysts, adsorbents, membranes, or electrodes, enabling the selective capture and conversion of CO_2_ into valuable products or its safe sequestration. The search for innovative materials with enhanced properties has intensified in recent years, driven by the urgency to find sustainable solutions for CO_2_ mitigation.^5^

In the field of DAC, materials capable of selectively adsorbing and absorbing CO_2_ from ambient air are highly desirable. These sorbents should possess a high affinity for CO_2_, excellent selectivity over other gases, a high adsorption capacity, and good stability. Metal–organic frameworks (MOFs), zeolites, and amine-functionalized materials, oxides, and hydroxides have shown promising results in CO_2_ capture from air. However, there is still a need for advanced materials with improved performance and lower energy requirements.^1,2^

In this field, the direct conversion of captured CO_2_ to valuable products is an excellent strategy to overcome some regeneration problems. CO_2_ valorization technologies aim to transform this gas into chemicals, fuels, or other value-added materials, thereby closing the carbon cycle. Catalysts are key components for those processes, as they facilitate the activation and transformation of CO_2_ molecules. In this field, various types of catalysts, including homogeneous, heterogeneous, electro and photocatalysts have been explored.^6,7^ Advancements in materials science have led to the development of novel catalysts with improved activity, selectivity, and stability. However, limited examples using atmospheric air to directly reuse the CO_2_ are observed. Thus, far, the products observed from the reutilization of atmospheric CO_2_ include cyclic carbonate,^8^ CO,^9^ formate,^10−12^ methanol,^13−16^ and more recently methane.^7^

Accordingly, this Perspective aims to provide a comprehensive overview of the recent advancements in materials for DACC. It will discuss the key materials used in DAC systems as well as the catalysts employed for the conversion. The focus will be on highlighting the performance, limitations, and prospects of these materials in the context of sustainable CO_2_ mitigation strategies. This paper will be organized into the subsequent sections: *(i) Exploration of materials for DAC; (ii) examination of materials and reactions in DACC; and (iii) future perspectives and concluding remarks*.

## 2 Materials for Direct Air Capture

Given the relatively low concentration of CO_2_ in ambient air, approximately 425 ppm (0.04%), the energy consumption of sorbents tends to be high. This is largely dictated by the type of material used, its sorption capacities, and selectivity toward the CO_2_. As such, the appropriate choice and development of sorbents are deemed crucial for effective DAC applications.^1−3^ The mechanisms for capturing CO_2_ predominantly involve *adsorption* and *absorption*. Adsorption is a surface phenomenon where the adsorbate, here, CO_2_, adheres to the surface of an adsorbent either via weak van der Waals forces (physisorption) or through stronger chemical bonds (chemisorption). Absorption, conversely, is a volume-based process where the absorbate permeates and uniformly disperses throughout the volume of the absorbent.^17^ A more encompassing term is *sorption*, which includes both adsorption and absorption. In this context, the terms *’sorption’* and *’sorbent’* will be used as the standard terminology.

**Figure 1:**
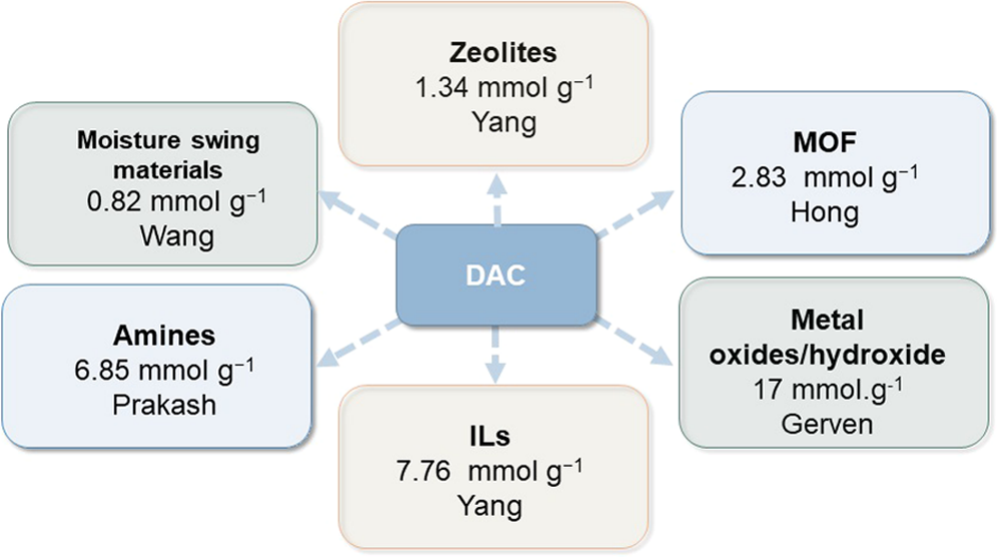
State-of-the-art materials for DAC.

Numerous sorbents, such as zeolite, MOF, and alkali oxides, have been extensively studied. Sorbents are generally categorized into physical and chemical types depending on their respective sorption mechanisms. Physisorption entails a lower energy cost for separating CO_2_ from the sorbent but involves higher expenses for activating and reusing the gas. The carbon atom of the CO_2_ molecule is sp hybridized, and the strong overlap of the bonding orbitals limits reactivity. In contrast, chemically captured CO_2_ adopts a more reactive configuration (trigonal planar sp^2^ hybridization) in the form of bicarbonate and carbamates. Between the both, the bicarbonate (−45 kJ/mol) is more reactive than carbamate (−80 kJ/mol) facilitating the reuse,^18^ suggesting that the formation of bicarbonate is a way to activate the CO_2_.^19^ This section provides a summary of the state-of-the-art sorbents for DAC (Figure 1).

### MOFs

MOFs have attracted significant attention in the field of DAC due to their unique properties. They are highly porous materials composed of metal ions coordinated with organic ligands. Their large surface areas and tailored pore sizes make them ideal candidates for efficient CO_2_ adsorption. The selective capture of CO_2_ from ambient air requires adsorbents with high affinity, excellent selectivity over other gases, high adsorption capacity, and good stability. MOFs can meet these requirements, and their performance can be further enhanced by incorporating specific functional groups, mainly amines, to improve the CO_2_ affinity and selectivity. Regarding the metal center, while Cu, Zn, Ni, Co, and Mg have been tested, the most promising results were obtained with Mg.^20−22^ Despite the great advance in this area, the state-of-the-art DAC using MOF is around 2.83 mmolCO_2_/g_mat_.^21,22^ This result was achieved using a diamine-functionalized MOF, en-Mg_2_(dobpdc) (en = ethylenediamine; dobpdc = 4,4′-dioxi-dobiphenyl-3,3′-dicarboxylate). The primary hurdle associated with MOFs is that their predominant mechanism of capture is physisorption, which inherently restricts the quantity of CO_2_ that can be absorbed.^21^

### Zeolites

Zeolites are crystalline aluminosilicate materials known for their well-defined nanoporous structures. Their unique pore sizes and shapes make them suitable for selective CO_2_ adsorption. Amine-modified zeolites, in which amine groups are grafted onto the zeolites, exhibit enhanced CO_2_ affinity and selectivity, making them promising materials for DAC systems. By tailoring the properties of zeolites, improved performance in terms of the CO_2_ capture efficiency has been observed. Yang and co-workers focused on optimizing zeolite structures and developing tailored synthesis methods to meet the requirements of large-scale DAC applications, resulting in sorption capacity of 1.34 mmolCO_2_/g_mat_.^23^ Currently, numerous researchers have presented a variety of zeolites that exhibit improved CO_2_ capture performance, accomplished through cation exchange and amine modification processes. However, the presence of water vapor is a significant factor impacting the CO_2_ adsorption efficacy of zeolites, as it can compete with CO_2_ for the active adsorption sites, which limited the use of these materials to capture CO_2_ directly from the air.^24^

### Amine-Functionalized Materials

Amines, characterized by their strong affinity for CO_2_, have been widely studied for CO_2_ capture applications, especially aqueous solutions of primary and secondary amines, such as mono- and diethanolamine. They react with CO_2_ to form carbamates, which can further transform into bicarbonate species in the presence of water. However, they are typically only used in 20–30% concentration in water due to corrosion and degradation issues. A significant downside of these solution-state CO_2_ capture methods is their high heat capacity, making the regeneration step energy-intensive and expensive. Furthermore, these amines are better suited for capturing CO_2_ from oxygen-free or low-oxygen gas mixtures as they tend to degrade over time. To mitigate energy costs, amines and polyamines on solid supports have been suggested as alternatives. Amine-functionalized materials, such as modified silica, polymers, and solid sorbents, chemically react with CO_2_ to form stable carbamate compounds. This chemisorption process enables efficient CO_2_ capture from the air, thus far yielding a sorption capacity of 6.85 mmolCO_2_/g_mat_. However, challenges associated with the regeneration of amine-based sorbents and their susceptibility to degradation remain areas of active research.^13,25^ The development of stable and regenerable amine-based materials is crucial for the practical implementation of DAC technologies.

### Metal Oxides and Hydroxide

Metal oxides, such as calcium oxide (CaO) and magnesium oxide (MgO), offer an alternative approach to CO_2_ capture. These materials capture CO_2_ through the formation of carbonates, which undergo reversible reactions under specific conditions. This reversibility allows for the release of captured CO_2_ for storage or utilization. Metal oxide-based sorbents have shown promise in terms of their capacity for CO_2_ capture and subsequent release, making them potential candidates for DAC systems. These materials exhibit remarkable sorption capacity (17 mmol of CO_2_/g_mat_), particularly due to their low molecular weight and basicity, which enables them to produce metal carbonates. However, challenges exist in terms of the energy requirements for regeneration and the optimization of capture/release cycles.^26^

Alkali hydroxide solutions also present remarkable interest for CO_2_ scrubbing, especially focusing on the further reuse of CO_2_. Recent reports in this regard have been published by Prakash and co-workers.^14,15^ The true value of this methodology stems from the fact that the hydrogenation of ester and bicarbonate intermediates, are significantly more efficient than that of formamide or carbamate intermediates resulting in the reaction between CO_2_ and amines.^14^

### Ionic Liquids (ILs)

ILs are described as organic salts with melting points below 100 °C and are predominantly solids at room temperature. Their potential in DAC is based on their versatility to combine various cations and anions, which offers the possibility of fine-tuning the chemical and physical attributes of the absorbent. Furthermore, particularly given their low volatility, minimal corrosiveness, exceptional chemical and thermal stability, nonflammable nature, and reduced vapor pressure are key features for sustainable CO_2_ capture process.^27,28^ In addition, basic ILs can activate water molecules, forming a guest@host complex that can react with CO_2_ to produce bicarbonate.^29^ This presents a significant advantage compared to other materials, as they can achieve a high sorption capacity even under humid conditions. Yang and co-workers showed that using atmospheric CO_2_ conditions pyrrolidinium-based IL with a borohydride anion was able to capture 7.76 mmol CO_2_/g_IL._^30,31^ The authors demonstrate not only the capture but the possibility to transform this CO_2_ into formate.^31^

### Moisture Swing Materials

Moisture swing materials have properties that change with the presence or absence of moisture, allowing them to alternate between absorbing and releasing CO_2_. This “moisture swing” between dry and wet states drives the cyclical process of CO_2_ capture (in the dry state) and release (in the wet state). In the dry state, the sorbent exhibits an affinity for CO_2_, allowing them to capture the gas from the surrounding environment. This is achieved through the formation of weak chemical bonds between the sorbent material and the CO_2_ molecules. Once the sorbent has reached its CO_2_ saturation point, the introduction of moisture alters the physical properties of the sorbent. The presence of water molecules disrupts the sorbent-CO_2_ interaction, leading to the desorption or release of previously captured CO_2_.

Unlike other carbon capture methods, such as thermal or pressure swing adsorption, which necessitate substantial energy input in the form of heat or pressure alterations, the moisture swing process capitalizes on ambient changes in humidity, thereby presenting a potentially energy-efficient and environmentally benign alternative. However, so for limited sorption capacity has been demonstrated (0.82 mmol CO_2_/g_mat_).^32^

### Others

Porous carbon materials, such as activated carbon and carbon nanotubes, have shown promise in DAC applications. These materials possess high surface areas and porosities, enabling them to absorb CO_2_ molecules through physical adsorption. However, challenges remain in terms of optimizing adsorption capacity and reducing energy requirements for regeneration processes.^1,2^

While substantial progress has been made in the field of DAC, these materials still present significant challenges. Specifically, their high energy demands, particularly for the regeneration process, pose a considerable problem. This process accounts for approximately 70% of the total budget of a CO_2_ capture system, as it requires temperatures exceeding 100 °C.^28^

## 3 Direct Air Capture and Integrated CO_2_ Conversion

The catalysts are a central point to transform the CO_2_ into added-value products. While there have been significant advances in materials science leading to the creation of improved catalysts primarily for pure CO_2_, there is limited work on using atmospheric CO_2_.^2,10−14,30,33^

**Figure 2:**
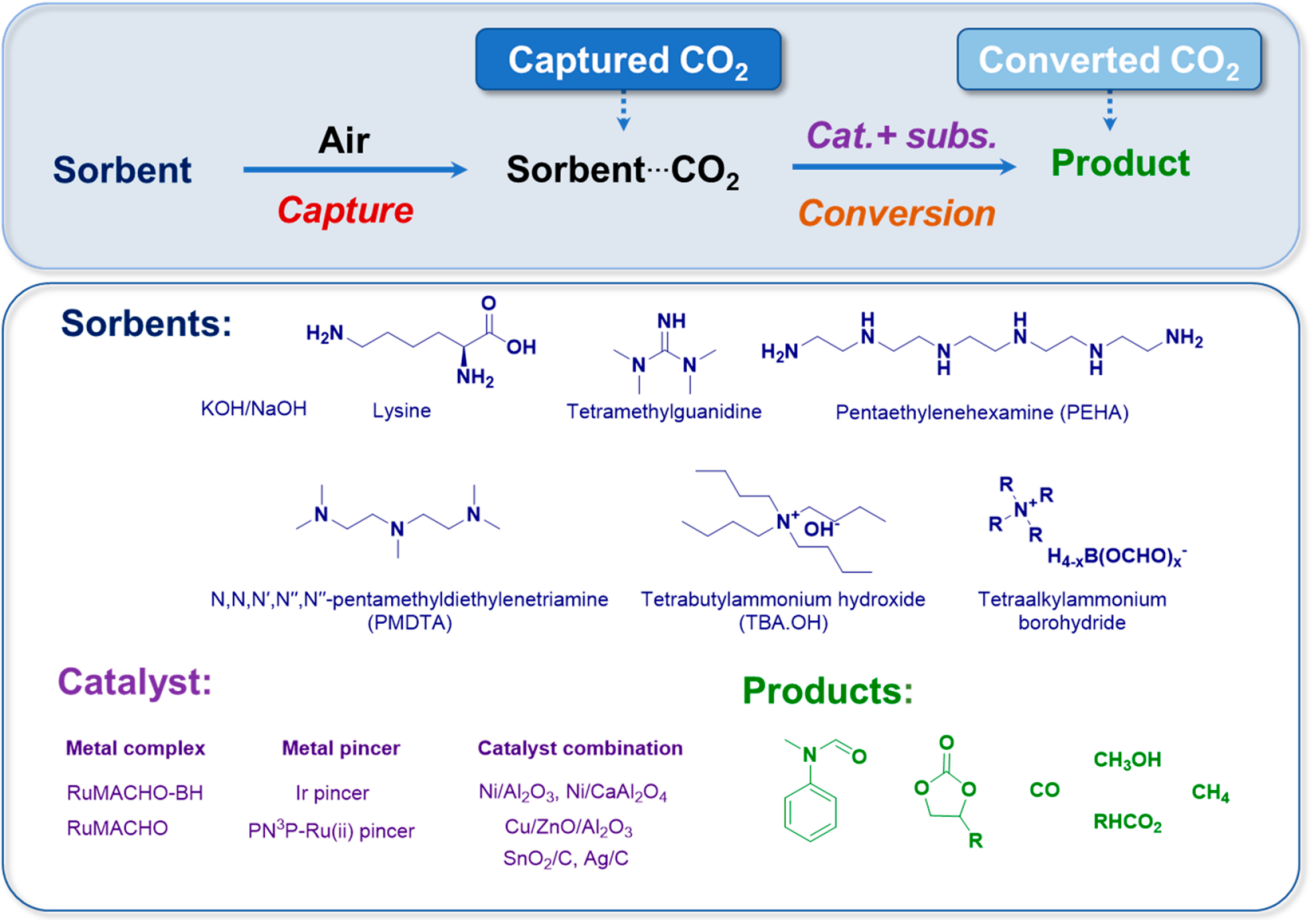
Generic route for DACC is described in the literature.

The pursuit of developing a singular material with the combined abilities of simultaneous or sequential sorption and catalysis for both CO_2_ capture and conversion is undeniably appealing, yet it presents considerable challenges. The process of extracting CO_2_ directly from the atmosphere holds significant climate-related advantages, but it is accompanied by substantial costs, as does the subsequent sequential conversion.^33^ To date, the examples of DACC are based on two-step reactions, first performing the capture and second, the conversion (Figure 2), yielding: cyclic carbonate,^8^ CO,^9^ formate,^10−12^ methanol^13−16^ and methane.^7^ In this section, each of these products will be described in detail.

### Cyclic Carbonate

The formation of cyclic carbonate using CO_2_ captured from air has been a focus of attention recently. In a pioneering work, Mg(II)-based MOFs demonstrated an efficient catalyst for directly converted CO_2_ from the atmospheric air into cyclic carbonates under mild conditions (60 °C, 48 h, balloon loaded with air), resulting in 92% of conversion for epichlorohydrin (ECH).^34^

More recently, our research group presented a groundbreaking methodology for DACC that enables the efficient transformation of atmospheric CO_2_ into cyclic carbonates. This novel technique leverages readily available basic ILs, eliminating the requirement for complex and expensive cocatalysts or sorbents, while operating under mild reaction conditions. Our methodology demonstrates exceptional performance, with the IL solution efficiently capturing CO_2_ from the atmospheric air (0.98 mol CO_2_/mol_IL_ by bubbling air with 425 ppm of CO_2_), and subsequently achieving complete conversion into cyclic carbonates using substrates derived from epoxides or halohydrins, potentially sourced from biomass. Mild condition was employed in this work, 40 °C; atmospheric CO_2_ (0.04%) and 16 h, resulting in >99% yield.^8^

### Formate

In 2018, Guan et al. introduced the use of Ru(II) PN^3^P pincer complexes for the purpose of hydrogenating CO_2_. These complexes exhibit remarkable selectivity and catalytic activity, with a high turnover frequency (TOF) of up to 13 000 h^–1^ and a turnover number (TON) of up to 33000, particularly in a biphasic system comprising tetrahydrofuran (THF) and water. One notable achievement of the study is the successful conversion of carbon dioxide from air into formate (69%) in the presence of an amine at 110 °C and 27 bar of H_2_. Importantly, the catalytic system employed in this study combines the advantages of both homogeneous and heterogeneous systems. This process enables separation of the product and recycling of the catalyst.^11^

Beller’s group, in 2021, presented an amino-acid-based reaction system for DACC to generate formates. The system incorporates naturally occurring amino acid l-lysine. By employing a specific Ru-based catalyst, mainly RuMACHO-BH, 80 bar H_2_, 145 °C, they achieved good activity by converting 46% of captured CO_2_ into formate, and TON > 50 000.^10^

In 2022, Choudhury and co-workers developed an efficient catalytic system based on phosphine-free Ir(III)-NHC (N-heterocyclic carbene) for DACC to generate formate. Tetramethylguanidine was used as a capturing agent, followed by conversion to formate using H_2_ gas (25–40 bar, 120 °C), and both steps were conducted in water. The system demonstrates impressive product yields of up to 85% and TON around 19 000 in 12 h of reaction. The utilization of a phosphine-free Ir(III)-NHC catalyst in this system offers a promising alternative for efficient and sustainable CO_2_ utilization.^12^

### Methanol

The process of creating methanol can entail the hydrogenation of CO_2_, in which CO_2_ undergoes a sequence of reactions to be transformed into methanol. Various catalysts and methods have been studied for this process. Commercial catalysts often consist of CuO and ZnO supported on Al_2_O_3_ with stabilizing additives and promotors. Amine and hydroxide solutions have been used for CO_2_ capture and in situ hydrogenation to methanol, allowing for the separation of amine and catalyst after the reaction.^13,35^

In 2016, Prakash and co-workers introduced significant advancements in the field of CO_2_ conversion. They demonstrated for the first time the development of a methodology for DACC to produce methanol from atmospheric CO_2_, achieving a yield of 79%. The catalyst system utilizes pentaethylenehexamine (PEHA) and Ru-MACHO-BH in a solvent, operating at temperatures ranging from 125 to 165 °C and a H_2_ pressure of 50 bar. The methanol separation from the reaction mixture was demonstrated through distillation. The catalyst could be recycled over five runs without significant loss of activity; however, the sorbent was not.^13^ This work initiates a string of publications on this subject from this research group.

In 2018, following capture in an aqueous amine solution, CO_2_ from the air was converted to methanol in a high yield (89%) within a MeTHF/H_2_O biphasic system. This system also facilitates separation and partial recycling of both the amine and the catalyst for multiple reaction cycles, retaining 87% of the initial cycle’s methanol productivity. The method consists of the use of Ru-MACHO-BH as catalyst and the polyamine PEHA as a sorbent.^36^

Another significant breakthrough was achieved in 2020 by the same group, with the establishment of the first alkali hydroxide-based system for capturing CO_2_ from the air and converting it into methanol. The study demonstrates that bicarbonate and formate salts can be efficiently hydrogenated to methanol with high yields in a solution of ethylene glycol. The researchers developed an integrated one-pot system, where CO_2_ is captured by an ethylene glycol solution containing the alkali hydroxide base. Subsequently, the captured CO_2_ is hydrogenated to produce methanol using Ru-PNP catalysts, performed at 140 °C and 70 bar H_2_. The resulting methanol was separated from the reaction mixture through distillation. Notably, the study also observed partial regeneration of hydroxide bases at low temperatures, which was an advance from previous works. The researchers suggest that the high capture efficiency and stability of hydroxide bases make them superior to the existing amine-based routes for DACC to methanol. They propose that this novel approach using hydroxide bases could be implemented in a scalable process for efficient and sustainable CO_2_ capture and methanol production.^14^

The same group demonstrated a similar system for methanol production using a heterogeneous commercial Cu/ZnO/Al_2_O_3_ catalyst. Among the evaluated solvents, glycols demonstrated a notable effect in promoting methanol formation at a temperature range of 170–200 °C utilizing molecular H_2_. Methanol yields of up to 90% were achieved. The catalytic process and potential reaction pathways were examined through control experiments, suggesting that hydrogenation in the presence of an alcohol proceeds through the formation of a formate ester as an intermediate. Lastly, a demonstration of DACC was showcased as a novel process for methanol synthesis, utilizing the combination of heterogeneous catalysis and air as a renewable carbon source.^15^

### Methane

Recently, DACC into methane has been first reported, with yields reaching up to 100% using both Ni/Al_2_O_3_ and Ni/CaAl_2_O_4_ catalysts. The methodology is based on the formation of metal carbonate through the sorption of CO_2_ from air into inorganic hydroxide. The conversion step was performed under 50 bar of H_2_, 48 h, and 225 °C. The authors demonstrated that water enhances the conversion of the carbonate salt to methane. The Ni/Al_2_O_3_ catalyst preserved 99% of its activity in the alkaline medium after five consecutive hydrogenation cycles^29^

### Other Chemicals

A proof-of-concept experimental setup where CO_2_ is captured from air in the form of a carbonate/bicarbonate solution via direct air capture and then converted to formate and CO, has been demonstrated by Breugelmans and co-workers. The findings presented open a new possibility for scaling up the electrochemical conversion of CO_2_.^9^

Lombardo et al. have demonstrated the capability of tetraalkylammonium borohydrides to effectively capture substantial amounts of CO_2_ and convert it into formic acid and N-formylated compound under ambient conditions. Their study revealed that these materials exhibit impressive CO_2_ absorption capacities since each BH_4_^–^ anion could react with three CO_2_ molecules, resulting in the formation of triformatoborohydride ([HB(OCHO)_3_]). The researchers accomplished direct capture and reduction of CO_2_ from the air using various tetraalkylammonium borohydrides, including tetraethyl, -propyl, and -butylammonium borohydrides. Interestingly, they observed that the alkyl chain length in these compounds played a significant role in the reaction kinetics and thermodynamics. Additionally, they achieved the transfer of formate for the N-formylation of an amine.^30^

**Figure 3:**
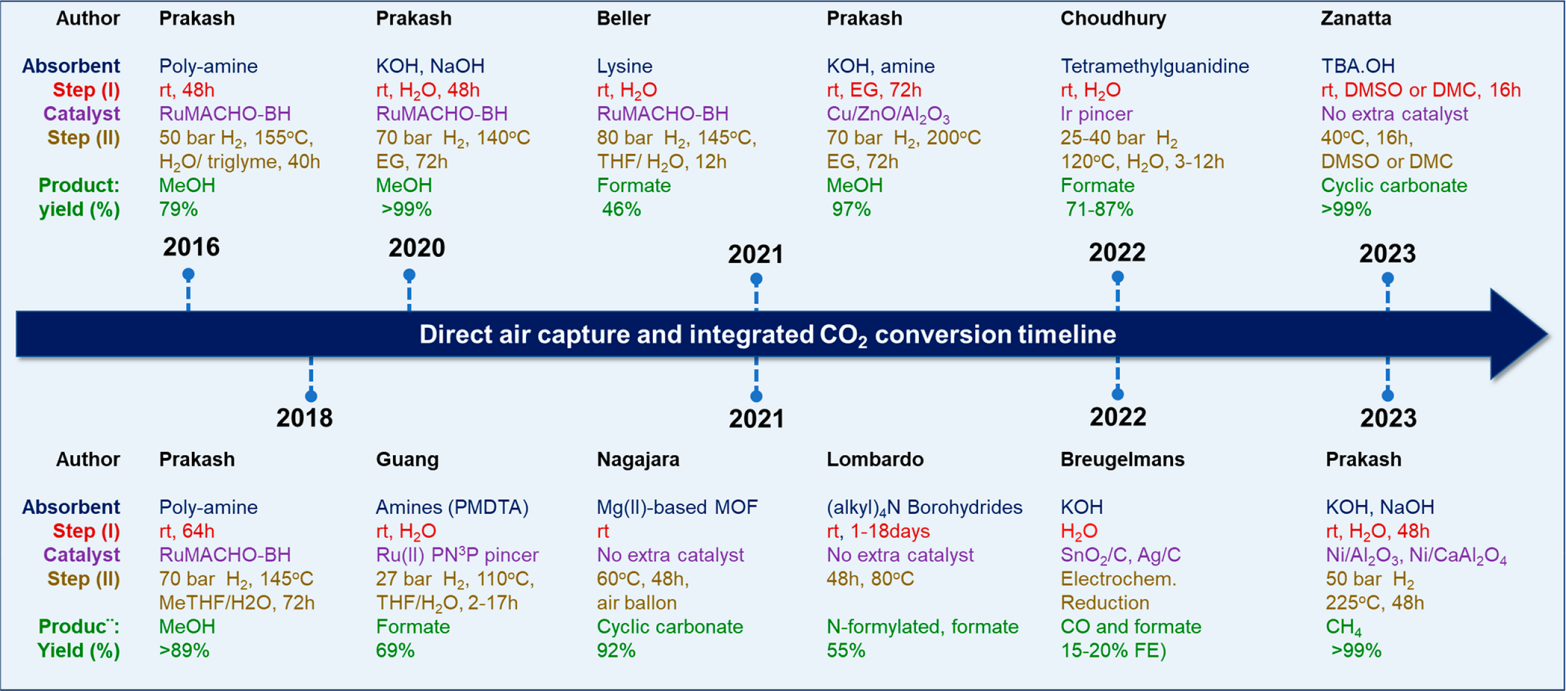
State-of-the-art in DACC.

Figure 3 demonstrates a summary of the reports related to the DACC mentioned above.

## 4 Challenges and Perspectives

To enable the practical implementation of DACC technologies, the development of materials should consider factors such as scalability, cost-effectiveness, and environmental sustainability. Materials with abundant and low-cost precursors offer advantages in terms of the cost reduction and availability for large-scale deployment. Furthermore, environmentally friendly synthesis methods that minimize energy consumption and waste generation are essential for sustainable materials production. The integration of these considerations into materials design and synthesis processes will contribute to the viability and widespread adoption of DACC technologies.

By capturing atmospheric CO_2_, the goal is to create *neutral emissions* and *lose the carbon cycle by producing synthetic fuels*. CO_2_ must be constantly removed from the atmosphere, oceans, and terrestrial biomass, thereby reducing excess CO_2_ and helping to mitigate climate change. The captured CO_2_ can be used as a feedstock for various carbon-based materials. It can be used in the production of cement, plastics, carbon fibers, and other industrial applications. This helps keep carbon stored in the infrastructure, preventing its release into the atmosphere for extended periods.

**Figure 4:**
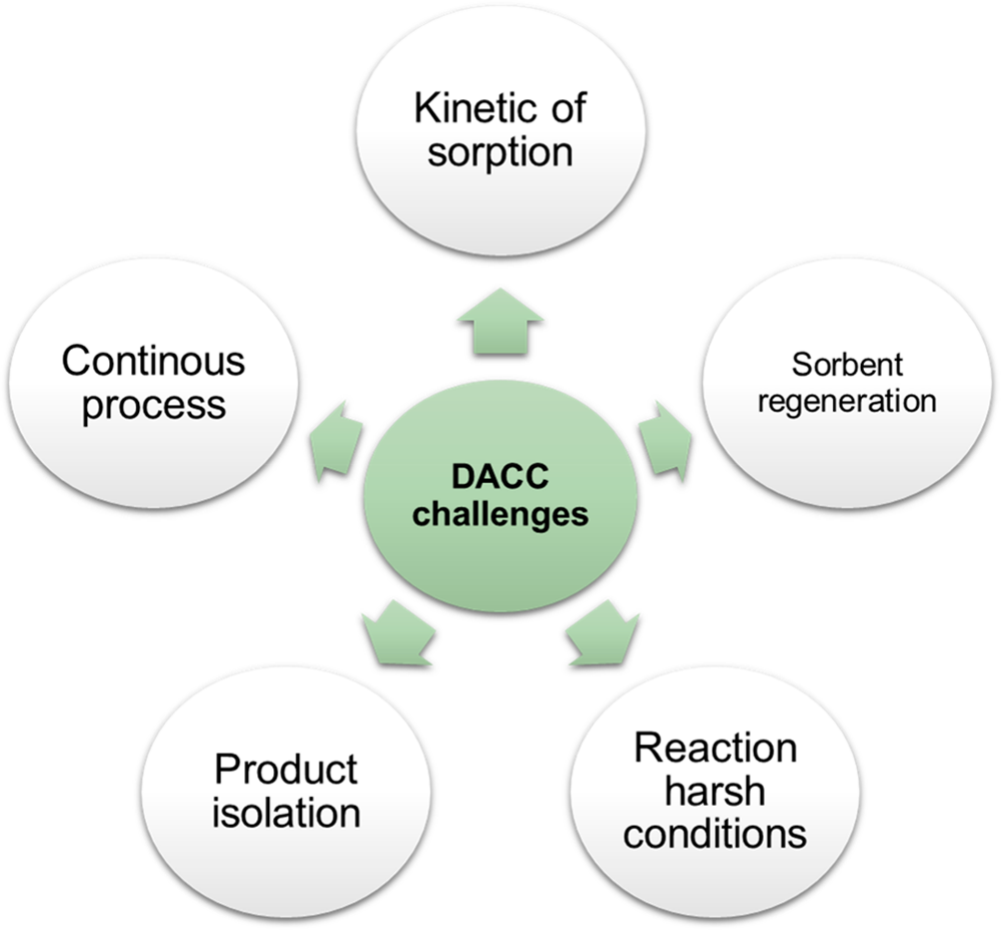
Critical challenges for DACC implementation.

However, for this to transition from concept to reality, the DACC must transpose five critical challenges (Figure 4):

1. *Slow kinetics of sorption*: When contrasting the capture process using pure CO_2_ with that of atmospheric CO_2_, the total time can stretch from under 15 min^37^ to a lengthy 16 to 72 h,^8,13,36^ respectively. In the combination of capture and conversion, the rate-limiting step is the initial sorption, especially when it is arduous to capture the gas from such a diluted source, as the atmospheric air that contains around 0.04% of CO_2_. A significant advancement lies in the development of materials and processes that can reduce this time. This necessitates further studies in this field to comprehend the process better and propose innovative alternatives.
2. *Efficient and cost-effective sorbent regeneration:* Typically, high energy is required, particularly for the regeneration process, which accounts for approximately 70% of the total budget of a CO_2_ capture system, given the necessity for temperatures exceeding 100 °C.^28,38^ Within this realm, the direct conversion of captured CO_2_ into valuable products emerges as a superb strategy to sidestep the energy costs associated with desorption and compression, thereby facilitating the closure of the carbon cycle. However, the existing examples are limited by the partial regeneration of the sorbent, which reduces around 10% of its capacity in each cycle.
3. *Rigorous conditions for converting CO_2_ into value-added products*: This process presents its own set of demanding conditions, requiring prolonged durations (12–72 h), elevated temperatures (110–200 °C), and substantial H_2_ pressures (25–80 bar).^7−16^ These stringent parameters pose challenges in terms of operational feasibility and safety. Maintaining high temperatures and pressures over extended periods not only increases energy demands but also heightens the risk of system failures and potential hazards. Therefore, developing techniques that can convert CO_2_ under milder conditions is still a challenge.
4. *Product isolation*: The most efficient catalysts reported to date for the DACC process are homogeneous, which complicates the final separation of the formed product. A strategy that has been employed is the use of a biphasic system, which can aid in catalyst regeneration.^13,14,36^ However, in certain instances, such as the production of formate, separation of the product remains a challenging task.^10^ Another alternative is the distillation process for product isolation, as demonstrated in the case of methanol. While this method is effective, it is also energy-intensive, posing an additional challenge to overall process efficiency.
5. *Scalable and continuous process*: Lastly, for DACC process to become a part of everyday reality, a substantial challenge lies in developing a process that can be easily scaled up or modified to fit existing industrial plants or even everyday applications. A scalable process would allow for greater capacity and flexibility, facilitating the integration of DACC technology into diverse sectors. Furthermore, the process needs to operate continuously to constantly capture CO_2_ from the atmospheric air and convert it into useful products. The continual operation would ensure a steady and reliable output, crucial for meeting ongoing demands and achieving the desired impact in terms of carbon capture and sequestration.

## 5 Conclusion

The development of advanced materials holds great promise for enhancing the efficiency and viability of DACC technologies. Through the integration of materials science advancements, researchers have made significant progress in developing adsorbents, membranes, sorbents, and catalysts with improved performance for CO_2_ capture and conversion. To date, sophisticated and expensive sorbents and catalysts have been necessary for these tasks as well as the limited number of added-value products generated. Further innovation in this field is crucial for the development of multifunctional materials capable of capturing, activating, and transforming CO_2_. This progress requires an interdisciplinary approach, particularly in integrating insights from chemistry, materials science, and engineering.
